# The role of Bacillus Calmette-Guérin administration on the risk of dementia in bladder cancer patients: a systematic review and meta-analysis

**DOI:** 10.3389/fnagi.2023.1243588

**Published:** 2023-08-24

**Authors:** Chao Han, Juan Wang, Ya-Li Chen, Cui-Ping Guan, Yan-An Zhang, Mao-Shui Wang

**Affiliations:** ^1^Department of Outpatient, Shandong Mental Health Center, Jinan, China; ^2^Department of Geriatrics, Shandong Mental Health Center, Jinan, China; ^3^Department of Lab Medicine, Shandong Public Health Clinical Center, Shandong University, Jinan, China; ^4^Shandong Key Laboratory of Infectious Respiratory Disease, Jinan, China; ^5^Department of Cardiovascular Surgery, Shandong Public Health Clinical Center, Shandong University, Jinan, China

**Keywords:** Bacillus Calmette-Guérin, Alzheimer's disease, dementia, risk factor, hazard ratio

## Abstract

**Background:**

Previous cohort studies have found an association between Bacillus Calmette–Guérin (BCG) administration and incident dementia. In the systematic review and meta-analysis, we aimed to summarize the current evidence of the effect of BCG use on the risk of developing dementia.

**Methods:**

We searched six databases until 20 May 2023 for studies investigating the risk of dementia and BCG administration. Hazard ratios (HRs) and 95% confidence intervals (95% CIs) were pooled in the meta-analysis. Meta-regression, subgroup, and sensitivity analysis were conducted as well.

**Results:**

Of the 4,043 records initially evaluated, five articles were included for final analysis, with a total of 45,407 bladder cancer (BC) patients. All five studies were evaluated and rated as with high quality, and a low possibility of publication bias was indicated. A significant association between BCG and the incidence of dementia in BC patients was found in all five studies. Although a high heterogeneity (I^2^ = 84.5%, *p* < 0.001) was observed, the pooled HR was 0.55 (0.42–0.73), indicating that BCG exposure or treatment reduced the risk of incident dementia by 45%. Moreover, the sensitivity analysis showed good robustness of the overall effect with no serious publication bias.

**Conclusion:**

BCG administration is associated with a significantly lower risk of developing dementia. However, an epidemiological cohort is needed to establish a relationship between BCG use and incident dementia in the normal population. Once the relationship is confirmed, more people may benefit from the association.

**Systematic review registration:**

identifier: CRD42023428317.

## Introduction

Alzheimer's disease (AD) is the most common form of dementia, accounting for two-thirds of all dementia diagnoses (Jia et al., 2020). As an early stage of dementia, AD is known as a neurological brain disorder that could develop into progressive memory loss, loss of independence, significant comorbidities, and even death (Gaugler et al., 2022). Although a recent meta-analysis demonstrated that dementia incidence showed a decreasing trend, AD incidence did not decline (Gao et al., 2019). The US estimated healthcare costs related to AD in 2020 are estimated to be USD$ 305 billion, with the cost expected to increase to more than USD$ 1 trillion as the population ages (Wong, 2020). Fortunately, many novel mechanisms [such as vital transcription factors (Rai et al., 2021) and circular RNAs (Awasthi et al., 2018)] were revealed, and therapeutic agents [Berberine (Singh et al., 2019b, 2021b), phytoconstituents (Singh et al., 2021a)] were found, and these agents would be helpful to improve the outcome of such patients.

The pathogenesis of AD includes two key features: accumulation of amyloid β (Aβ) plaques and neurofibrillary tangles (hyperphosphorylated tau protein) (Kinney et al., 2018). Aβ and tau species could activate astrocytes and the brain's resident macrophages (microglia), which release many pro-inflammatory cytokines, such as tumor necrosis factor α (TNF-α), interleukin 1β (IL-1β), and oxidative stress biomarkers (Singh et al., 2019a; Novoa et al., 2022; Wiatrak et al., 2022). Then, neuroinflammation is triggered. However, this inflammatory response can lead to Aβ and tau overproduction and induce neurodegeneration, synapse damage, and neuronal death (Novoa et al., 2022; Wiatrak et al., 2022). In general, accumulating evidence supports that the development of AD is associated with the immune system. To date, several inflammatory mediators have been proposed as AD markers, such as TNF-α, IL-1β, ionized calcium-binding adapter molecule-1 (Iba-1), glial fibrillary acidic protein (GFAP), and nuclear factor kappa B (NF-κB) (Novoa et al., 2022). Moreover, a boost in peripheral immune cells can improve neurodegeneration by restoring the balance between the immune system and the brain (Baruch et al., 2015).

Bacillus Calmette–Guérin (BCG) is a live attenuated form of *Mycobacterium bovis* that was designed as a vaccine against tuberculosis. In practice, intravesical BCG is administered for preventing the recurrence of non-muscle invasive bladder cancer (NMIBC). Numerous meta-analyses have proved its efficacy (Chen et al., 2018). Although the immune effects of BCG have been recognized, the exact mechanism of anticancer activity is still unclear and may be explained as follows: it binds fibronectin in the bladder wall and stimulates Th1 cells to secrete multiple cytokines, such as interferon-γ, TNF-α, and ILs. Then, these cytokines induce cell-mediated cytotoxic activity in cancer cells (Stassar et al., 1994; Taniguchi et al., 1999).

Recently, some trials suggested that, in bladder cancer (BC) patients, BCG users have a decreased incidence of AD. However, these studies have some limitations, such as different diagnostic codes (Weinberg et al., 2023), selection bias (Gofrit et al., 2019a), different BCG strains (Klinger et al., 2021), and study design (Gofrit et al., 2019b; Kim et al., 2021; Makrakis et al., 2022). Hence, a systematic review is required to be performed to avoid such disadvantages. In the present report, we reviewed the most up-to-date evidence about the association between BCG administration and the risk of AD and other dementia in BC patients. Then, we tested whether intravesical BCG vaccine therapy for patients with BC is associated with a decreased risk of developing dementia. Subsequently, we further combined estimated hazard ratios (HRs) of incident dementia associated with BCG use.

## Methods

### Search strategy

The protocol for this study has been registered in the PROSPERO database (CRD42023428317). This meta-analysis was reported following the Meta-analysis Of Observational Studies in Epidemiology (MOOSE) guidelines (Stroup et al., 2000).

Databases including PubMed, Embase, Scopus, Web of Science, CINAHL, and Cochrane Library were searched for relevant studies published up to 20 May 2023. The search terms included but were not limited to the following: BCG, vaccine, AD, and dementia. The search strategy for each database was detailed in the Supplementary material.

### Study eligibility

Studies that evaluated the role of BCG administration on the risk of incident dementia were included. No specific criteria for the study design were given. The exclusion criteria of the study were as follows: duplicate, non-English document type (such as conference paper, review, book chapter, protocol, or comments), insufficient data, unavailable full text, animal studies, and bench studies.

Duplicates were removed by automated processing in EndNote. Then, two authors (CH and YL-C) screened titles and abstracts independently to identify relevant studies. Finally, the available full texts were systematically examined to evaluate compliance with our inclusion and exclusion criteria (CH and YL-C). When there were disagreements, a third reviewer (JW) made the final decision.

### Study selection and data extraction

Data were extracted from eligible studies by two independent investigators (CH and YL-C). Any disagreements were resolved through consultation with a third investigator (JW), if necessary. The following information was extracted from the included studies: first author, year of publication, study design, study period, region, sample size, gender, age, follow-up period, BCG interventions, dementia subjects, effect size (such as HR), and corresponding 95% confidence intervals (CIs).

### Quality assessment

The quality of the included studies was assessed using the Newcastle–Ottawa Scale (NOS) by two independent authors (CH and YL-C), and disagreements were resolved through a third reviewer (JW). The NOS assigns a maximum of 9 points based on the following items: selection, comparability, and outcome. Subsequently, the bias risk of each study was rated as high (≤4), medium (5–6), and low (7–9), respectively.

### Statistical analysis

We calculated the pooled effect size (HR) and the corresponding 95% CIs for incident dementia in the BCG group compared with the non-BCG group. The heterogeneity was quantified using the Q test and I^2^. If the heterogeneity is considered significant (I^2^ > 50% or *P* < 0.05), a random effects model would be employed. Otherwise, the fixed-effect model would be adopted. Subgroup analyses stratified by region, age, gender, race/ethnicity, dose, follow-up period, and outcome were performed to examine subgroup differences and potential heterogeneity sources. Sensitivity analyses were performed to assess the robustness of the primary results. Moreover, the results of the fixed effects and random effects models were compared to check the stability of the pooled results. The Harbord test and Egger's test were used to evaluate potential publication bias. All statistical analysis was performed using STATA (Version, 15.0; Stata Corporation, College Station, TX). A *p* < 0.05 was considered to be statistically significant.

## Results

### Literature selection

**Figure 1:**
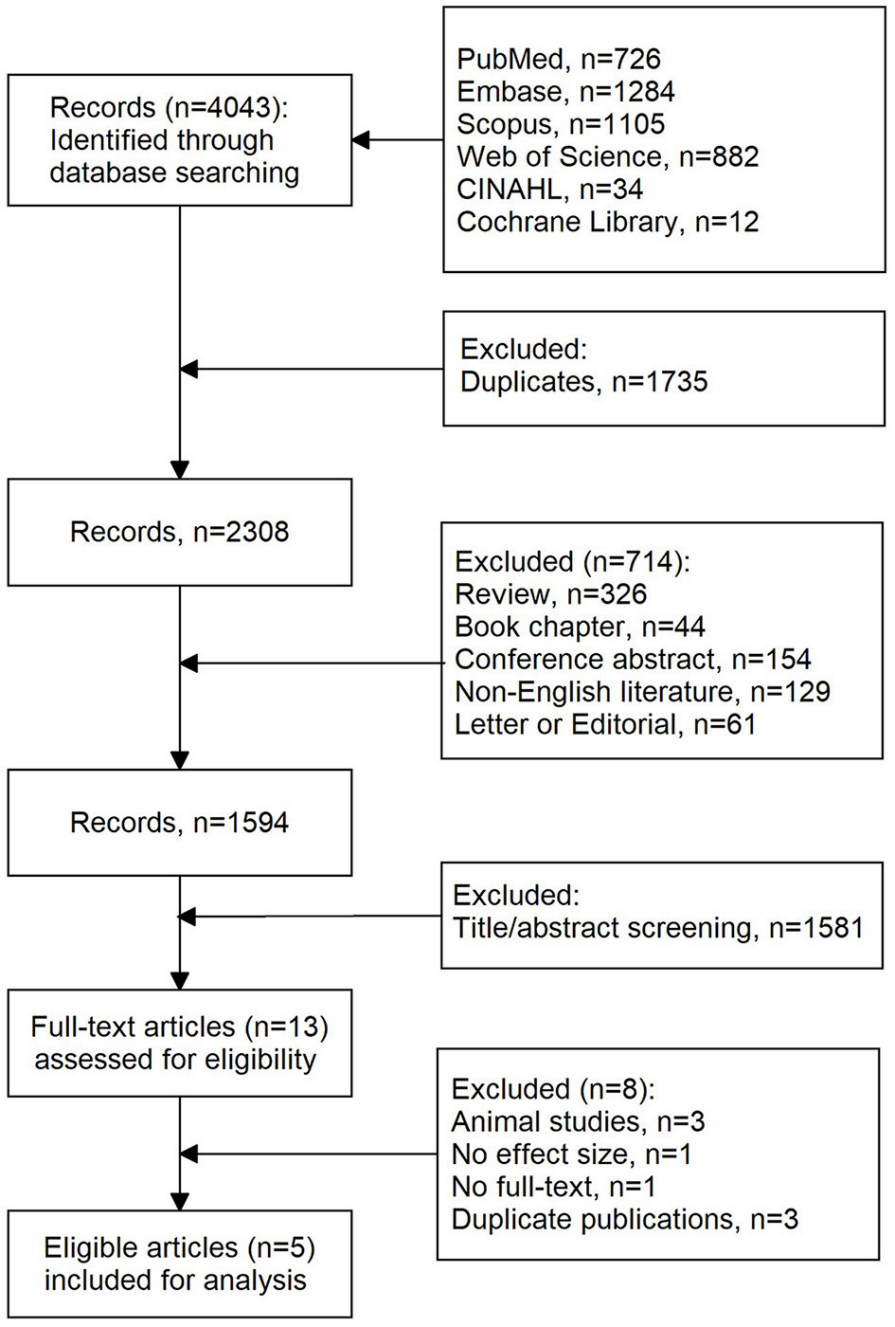
Literature selection process.

A total of 4,043 records were initially retrieved using our search strategy. As shown in Figure 1, after the removal of duplications (*n* = 1,735), 2,308 records were left. Of them, 714 records were further excluded due to reviews (*n* = 326), book chapters (*n* = 44), conference abstracts (*n* = 154), non-English literature (*n* = 129), and letters or editorials (*n* = 61). Then, 1,583 records were excluded after screening titles and abstracts. Eight studies were further excluded because of the following reasons: animal studies (*n* = 3), no effect size (*n* = 1), no full text (*n* = 1), and duplicates (*n* = 3). Finally, five eligible articles were included in this meta-analysis.

### Study characteristics

The characteristics of the included studies (*n* = 5) are summarized in Table 1. All studies had a retrospective nature, with a total number of 45,407 participants (sample sizes ranging from 700 to 26,584), and were published between 2019 and 2023. The studies were conducted in Israel (*n* = 2) or the USA (*n* = 3), respectively. The study period (duration from study entry date to study end) of each study ranged from 6 to 52 years, and the mean/median follow-up period varied from 3 to 8 years. All participants (experimental group) in these studies were BC patients receiving BCG induction or maintenance, while AD or other dementia patients were defined according to diagnostic codes and/or drugs prescribed. Among them, three studies considered incident AD as the outcome, and another two studies considered dementia (including AD) as the outcome. All studies were rated with the maximum score according to the NOS, indicating a high quality (Table 2).

**Table 1:** The characteristics of included studies.

**Study characteristics**					**Patient characteristics**									**BCG group**		**Non-BCG group**		**Cox proportional hazards regression analysis**				
**Sequence**	**First author, year**	**Study period**	**Study design**	**Region**	**Subjects**	**Sample size (n)**	**Female (n, %)**	**Age (mean** ± **SD)**	**Age (≥75 y, n, %)**	**Definition ofdementia**	**Definition of BCG Group**	**BCG Group (n, %)**	**Mean/median follow-up period (years)**	**Dementia**	**Non-dementia**	**Dementia**	**Non-dementia**	**Adjustedhazard ratio**	**95% CI (Lower)**	**95% CI (Upper)**	**Confoundingfactors**	***p*** **-value**
1	Gofrit et al., 2019a	1966–2018	Retrospective	Israel	BC patients underwent transurethral resection	1,371	237, 17.3%	68.1 ± 13.0	878, 64%	AD	BCG exposure or treatment (≥1 dose of BCG)	878, 64.04%	8	21	857	44	449	0.21	0.12	0.35	Tumor grade, stage, and gender	4.08^*^10–9
2.1	Klinger et al., 2021	2000–2019	Retrospective	Israel	BC patients from CHS, aged >60 years	6,725	1,167, 17.4%	73.7 ± 7.99	3,100, 46.1%	AD	BCG exposure or treatment (≥3 doses of BCG within a 120-day period)	1,578, 23.46%	6.6	75	1,503	336	4,811	0.79	0.61	1.01	Age at diagnosis and sex	0.062
2.2		2000–2019		Israel	BC patients from HUH, aged >60 years	700	116, 16.6%	74.2 ± 8.1	325, 46.4%	AD	BCG exposure or treatment (≥3 doses of BCG within a 120-day period)	408, 58.29%	5	13	395	17	275	0.25	0.12	0.54	Age at diagnosis and sex	0.001
2.3		2013–2019		USA	BC patients from UCLAH, aged >60 years	2270	590, 26.0%	70.3 ± 12.2	860, 37.9%	AD	BCG exposure or treatment (≥1 dose of BCG)	132, 6.02%	3.5	0	132	79	2,059	-	-	-	Age at diagnosis and sex	-
3.1	Kim et al., 2021	1984–2020	Retrospective	USA	NMIBC patients from MHS	1,290	380, 29.5%	71.5 ± 11.98	NM	AD or dementia	BCG exposure or treatment (≥1 dose of BCG)	319, 24.7%	3	10	309	89	882	0.41	0.21	0.80	Age, gender, race, and history of heart disease, cerebrovascular disease, and diabetes	0.009
3.2										AD	BCG exposure or treatment (≥1 dose of BCG)			2	317	26	945	-	-	-		-
4	Makrakis et al., 2022	2004–2015	Retrospective	USA	High-risk NMIBC from SEER-Medicare database, aged >66 years	26,584	5,773, 21.7%	78 ± 7.4	NM	AD	BCG exposure or treatment (≥1 dose of BCG)	13,496, 50.8%	3.25	964	12,532	1,228	12,130	0.73	0.67	0.79	Age, sex, race, T-stage, and CCI.	-
5	Weinberg et al., 2023	1987.5.28–2021.5.6	Retrospective	USA	NMIBC from RPDR, aged >50 years	6,467	1,686, 26.1%	70.3 ± 9.7	3,334, 51.6% (aged ≥70 y)	AD or dementia	BCG exposure or treatment (≥1 dose of BCG)	3,388, 52.38%	7	202	3,186	262	2,817	0.80	0.69	0.99	Age, sex, and CCI	0.02

BCG, Bacillus Calmette-Guérin; SD, standard deviation; 95% CI,95% confidence interval; BC, bladder cancer; AD, Alzheimer's disease; CHS, Clalit Health Services; HUH, Hadassah University Hospitals; UCLAH, UCLA Health System; MHS, Montefiore Health System; NMIBC, non–muscle-invasive bladder cancer; SEER, Surveillance, Epidemiology, and End Results; CCI, Charlson Comorbidity Index.

**Table 2:** The quality assessment of included studies.

**Study (cohort)**	**Representativeness of exposed cohort (1)**	**Selection of non-exposed cohort (1)**	**Ascertainment of exposure (1)**	**Outcome not present before study (1)**	**Comparability (2)**	**Assessment of outcome (1)**	**Follow-up long enough (1)**	Adequacy of follow up^*^(1)	**Quality score**
Gofrit et al. (2019a)	^⋆^	^⋆^	^⋆^	^⋆^	^**^	^⋆^	^⋆^	^⋆^	9
Klinger et al. (2021)	^⋆^	^⋆^	^⋆^	^⋆^	^**^	^⋆^	^⋆^	^⋆^	9
Kim et al. (2021)	^⋆^	^⋆^	^⋆^	^⋆^	^**^	^⋆^	^⋆^	^⋆^	9
Makrakis et al. (2022)	^⋆^	^⋆^	^⋆^	^⋆^	^**^	^⋆^	^⋆^	^⋆^	9
Weinberg et al. (2023)	^⋆^	^⋆^	^⋆^	^⋆^	^**^	^⋆^	^⋆^	^⋆^	9

^⋆^Follow-up period (median, ≥3 years; or maximum, ≥5 years) was considered enough. The quality of studies were assessed using the Newcastle–Ottawa (NOS) scale. The NOS scale is a star rating system that allocates a maximum of nine stars across three categories: participant selection (four stars; the first four columns, Column 2–5), comparability (two stars; Column 6) and measurement of outcome in cohort (three stars; Column 7–9). At last, the score (Column 10) of each study was calculated based on the summary. In addition, a high score means a low bias risk.

### BCG administration and incident dementia

**Figure 2:**
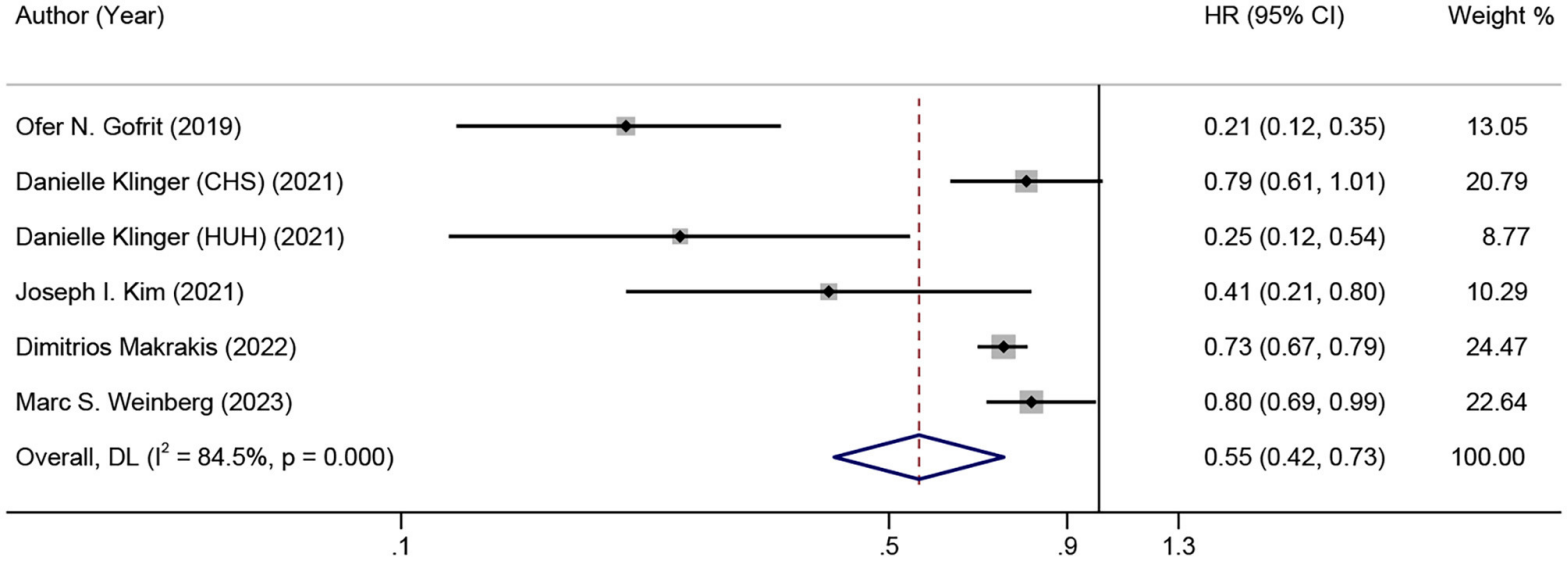
Forest plot for the association between BCG administration and incident dementia.

Figure 2 shows the combined effect estimate (HR) for the incidence of dementia in relation to BCG. A high heterogeneity (I^2^ = 84.5%, *p* < 0.001) was observed, so the random effects model was used. All studies showed a significant association between BCG and the incidence of dementia in BC patients, and the pooled HR was 0.55 [95% CI: 0.42–0.73, *p* (overall effect) < 0.001], indicating that BCG exposure or treatment reduced the risk of incident dementia by 45%.

### Subgroup analyses

Subgroup analyses were performed stratified by region, age, gender, race/ethnicity, dose, follow-up period, and outcome (Table 3). The study heterogeneity was associated with Israel (HR = 0.36, 95% CI: 0.13–0.96; *p* = 0.041), the USA (HR = 0.73, 95% CI: 0.63–0.85; *p* < 0.001), age (≥75 years: HR = 0.68, 95% CI: 0.62–0.75; *p* < 0.001), dose (≤6 doses: HR = 0.84, 95% CI: 0.75–0.94, *p* = 0.002; >6 doses: HR = 0.61, 95% CI: 0.46–0.82, *p* = 0.001), follow-up period (≤5 years: HR = 0.46, 95% CI: 0.23–0.89, *p* = 0.022; >5 years: HR = 0.55, 95% CI: 0.32–0.94, *p* = 0.03), and AD (HR = 0.47, 95% CI: 0.30–0.75, *p* = 0.001). Therefore, BC patients with such characteristics (from Israel, old age, high dosage, short follow-up period, and AD as outcome) may have a low risk of incident dementia.

**Table 3:** Subgroup analysis of the association between BCG administration and incident dementia.

**Subgroups**		**Studies (n)**	**Hazard ratio (95% CI)**	***p-*value_(overaleffect)_**	**Heterogeneity**		**Effects model**	***p*-value_(meta − regression)_**
					***I** ^2^*	***p***		
Region	Israel	3	0.36 (0.13–0.96)	0.041	91.7%	< 0.001	Random	0.337
	USA	3	0.73 (0.63–0.85)	<0.001	47.3%	0.150	Random	
Age	<75 years	2	0.80 (0.61–1.04)	0.114	51.5%	0.151	Random	0.289
	≥75 years	2	0.68 (0.62–0.75)	<0.001	0.0%	0.904	Fixed	
Gender	Male	2	0.58 (0.25–1.39)	0.223	74.7%	0.047	Random	0.955
	Female	2	0.62 (0.36–1.08)	0.092	0.0%	0.706	Fixed	
Race/ethnicity	Non-Hispanic black	2	1.67 (0.83–3.36)	0.151	85.7%	0.008	Random	0.112
	Hispanic white	2	1.13 (0.92–1.39)	0.244	25.9%	0.245	Fixed	
	Other	2	0.84 (0.68–1.04)	0.112	0.0%	0.567	Fixed	
Dosage	≤6 doses	2	0.84 (0.75–0.94)	0.002	43.7%	0.182	Fixed	0.461
	>6 doses	3	0.61 (0.46–0.82)	0.001	80.2%	0.006	Random	
Follow-up period	≤5 years	3	0.46 (0.23–0.89)	0.022	91.0%	<0.001	Random	0.463
	>5 years	3	0.55 (0.32–0.94)	0.03	80.3%	0.006	Random	
Outcome	AD	4	0.47 (0.30–0.75)	0.001	89.3%	<0.001	Random	0.607
	Dementia	2	0.62 (0.33–1.17)	0.142	72.0%	0.059	Random	
Total		6	0.55 (0.42–0.73)	<0.001	84.5%	<0.001	Random	

### Bias assessment and sensitivity analysis

**Figure 3:**
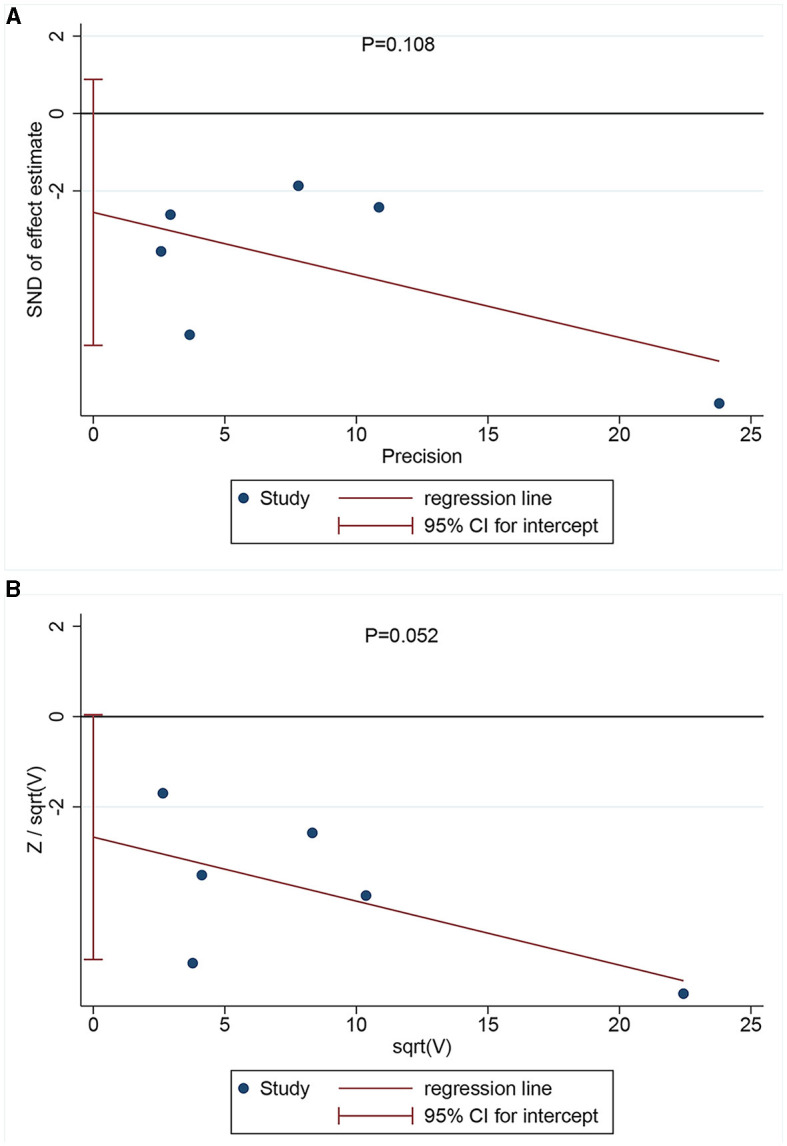
Publication bias assessment *via* Egger's **(A)** (*P* = 0.108) and Harbor's tests **(B)** (*P* = 0.052).

**Figure 4:**
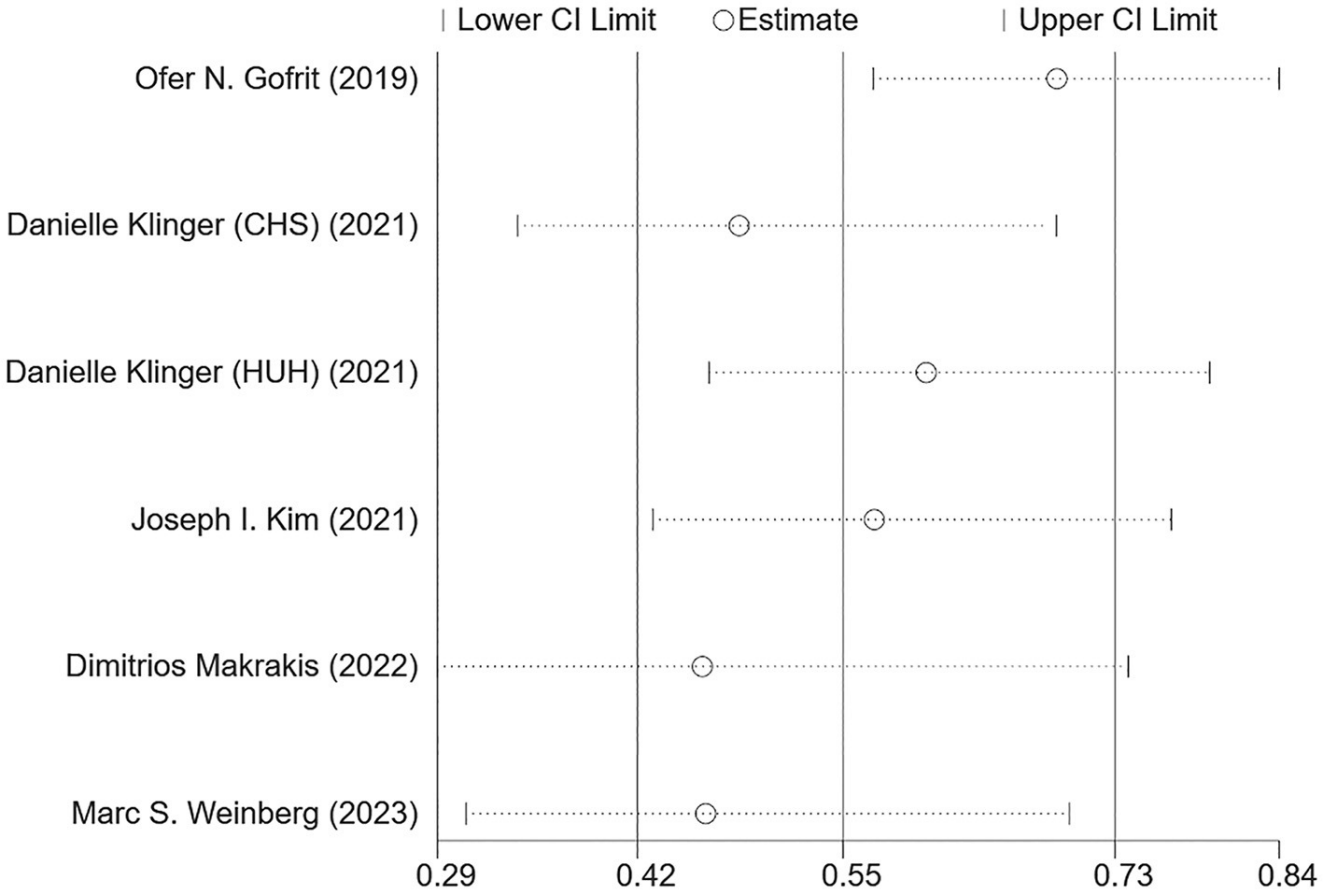
Sensitivity analysis of the association between BCG administration and incident dementia.

Egger's (Figure 3A) and Harbor's tests (Figure 3B) were performed for the assessment of publication bias, and the corresponding *p*-values were 0.108 and 0.052, respectively. The results indicated a low possibility of publication bias. Then, the stability of the meta-analysis results was assessed using the sensitivity analysis (leave-one-out method). When excluding any single study, there was no significant change in the pooled HR, indicating a robust result (Figure 4). Moreover, we further tested the stability of the results by comparing the results of the fixed and random effects models. The results remained stable in all groups, except in groups involving patients aged <75 years, male individuals, non-Hispanic black individuals, and patients with dementia, where their results shifted from a non-significant stage to a significant stage (Table 4).

**Table 4:** Comparison of the estimates by random-effects vs. fixed-effects models.

**Groups**		**Hazard ratios (95% CI)**	
		**Effects model (random)**	**Effects model (fixed)**
Region	Israel	0.36 (0.13–0.96)	0.58 (0.46–0.72)
	USA	0.73 (0.63–0.85)	0.74 (0.68–0.79)
Age	<75 years	0.80 (0.61–1.04)	0.74 (0.68–0.80)
	≥75 years	0.68 (0.62–0.75)	0.68 (0.62–0.75)
Gender	Male	0.58 (0.25–1.39)	0.77 (0.59–1.00)
	Female	0.62 (0.36–1.08)	0.62 (0.36–1.08)
Race/ethnicity	Non-Hispanic black	1.67 (0.83–3.36)	1.34 (1.11–1.61)
	Hispanic white	1.17 (0.87–1.59)	1.13 (0.92–1.39)
	Other race/ethnicity	0.84 (0.68–1.04)	0.84 (0.68–1.04)
Dosage	≤6 doses	0.76 (0.50–1.15)	0.84 (0.75–0.94)
	>6 doses	0.61 (0.46–0.82)	0.65 (0.59–0.72)
Follow-up period	follow- up ≤ 5 years	0.46 (0.23–0.89)	0.72 (0.66–0.78)
	Follow-up >5 years	0.55 (0.32–0.94)	0.72 (0.63–0.84)
Outcome	AD	0.47 (0.30–0.75)	0.71 (0.66–0.77)
	Dementia	0.62 (0.33–1.17)	0.76 (0.64–0.91)
Total		0.55 (0.42–0.73)	0.72(0.67–0.77)

## Discussion

In this systematic review and meta-analysis, the association between BCG use and incident dementia was investigated. Since only BC patients were included, the study overcame the health patient bias, which is a serious concern for most studies of vaccination and dementia risk. Our data demonstrated that patients not treated with BCG had a significantly higher risk of developing dementia. Although a significant heterogeneity (I^2^ = 84.5%, *p* < 0.001) was observed, a pooled HR was estimated at 0.55 (0.42–0.73), which indicated that BCG use could reduce the risk of incident dementia in BC patients. Moreover, according to meta-regression results, patients have the following characteristics, such as from Israel, old age, high dosage, short follow-up period, and AD as outcomes appear to have a reduced incidence of dementia after BCG vaccine treatment. Regarding the management of dementia, a dose-response relationship should be considered in practice, and BCG treatment may be more effective for AD than other types of dementia.

Due to the long-term non-specific immune effects of BCG exposure, a hypothesis that BCG could decrease the prevalence of AD in the elderly population was proposed (Gofrit et al., 2019a), which was investigated subsequently, and several claims were made previously. First, previous studies supported that BCG treatment is associated with a reduced risk of developing AD or other dementias (Gofrit et al., 2019b; Kim et al., 2021; Klinger et al., 2021; Makrakis et al., 2022; Weinberg et al., 2023). Second, old age was usually known as a risk factor for developing AD (Makrakis et al., 2022). Fortunately, BCG was effective for BC patients at any age to reduce the risk of incident AD (Gofrit et al., 2019a), especially in the elderly population (Klinger et al., 2021; Weinberg et al., 2023). Our study supports the idea that an older age appears to have a high non-statistical risk of incident AD and other types of dementia. Third, although a previous study found that sex distribution is associated with the prevalence of incident AD (Kim et al., 2021), our data do not support it. Although no significant variable was found in our meta-regression, patients with female sex and old age appear to have a lower incidence of dementia. Fourth, patients with high BCG exposure had a further lowered incidence of AD or another type of dementia than patients who had low BCG exposure (Kim et al., 2021; Makrakis et al., 2022), which was dose-dependent. Fifth, the role of BCG exposure in the prevalence of Parkinson's disease remains controversial due to a paradoxical response (Gofrit et al., 2019b; Klinger et al., 2021).

To date, the mechanism of association between BCG and dementia remains unclear and requires further investigation. Several potential mechanisms have been discussed previously: (1) intravesical BCG instillation may increase the immunosuppressive Treg population by IL-2 cytokines (Baek et al., 2016; Li et al., 2018), which could slow the development of clinical definite syndrome as in the case of multiple sclerosis (Sethi et al., 2014); (2) the BCG vaccination of adults could increase the glucose utilization (Kuhtreiber et al., 2018), which reverses cognitive aging (Minhas et al., 2021); and (3) the neuroinflammation of AD is primarily driven by the microglia (Gate et al., 2020). Neuroinflammation could be alleviated by recruiting inflammation-resolving monocytes to the brain, which was confirmed by the animal AD model (Zuo et al., 2017). In general, more evidence is required to clarify the precise mechanisms underlying the effects of BCG on neuroinflammation.

Recently, similar studies were evaluated for other vaccinations [such as influenza vaccination (Wiemken et al., 2021), herpes zoster vaccination (Scherrer et al., 2021b, 2022), and tetanus, diphtheria, and pertussis vaccination (Scherrer et al., 2021a)]. In a recent meta-analysis, all vaccinations (such as rabies, tetanus, diphtheria, and pertussis, herpes zoster, influenza, hepatitis A, typhoid, and hepatitis B) were associated with a trend toward reduced dementia risk, and an overall 35% lower dementia risk was reported (Itzhaki et al., 2020). Moreover, full vaccination types have a lower risk of developing dementia (Itzhaki et al., 2020; Wiemken et al., 2022). The role of influenza vaccination is also confirmed in another two meta-analyses, which suggests that it could reduce dementia risk significantly (Veronese et al., 2022; Sun et al., 2023). The vaccination against influenza may be helpful for the prevention of dementia, and subjects may benefit from more annual influenza vaccinations (Itzhaki et al., 2020). Interestingly, a paradoxical finding was observed for infections and a recent cohort (Douros et al., 2023). Infections, such as *Chlamydia pneumoniae*, human herpesvirus-6, Epstein-Barr virus, Herpes simplex virus-1, and the Herpesviridae family were found to be associated with a higher risk of AD (Ou et al., 2020). The association between infections and AD failed to gain substantial traction and was largely debated by the AD research community for many years (Itzhaki et al., 2020; Rai et al., 2022). More evidence for and against the infectious theory of AD should be weighed up, which may facilitate future research and drug development. Douros A et al. found that, although a decreased risk of dementia was observed for individual vaccines (shingles and diphtheria vaccines), all vaccines (such as influenza, pneumococcal, shingle, diphtheria, tetanus, and pertussis vaccines) were found with an increased risk of dementia [OR, 1.38 (95% CI, 1.36–1.40)], compared with no exposure (Douros et al., 2023). A further systematic review may be required to investigate the association between dementia risk and all vaccination.

The interpretation of this systematic review and meta-analysis must be considered within its limitations. First, all studies have a retrospective nature. Therefore, selection bias cannot be ruled out. Second, BC patients are indicated for BCG administration, and most BC patients have a good prognosis and long-term follow-up. The two key elements support testing the hypothesis in BC patients. However, the role of BCG administration remains unclear in the normal population, and further cohort studies are required. Third, although meta-regression results support the dose-dependent relationship between BCG use and the risk of dementia, the relationship was not evaluated in the study. Moreover, there was a lack of competing risk assessments. These were due to the limited availability of data from included studies. Finally, caution is required for the interpretation since a high heterogeneity exists in the analysis.

In conclusion, the present systematic review and meta-analysis indicates that BCG administration is associated with a decreased risk of dementia in BC patients. The findings suggest that the BCG administration may also have a preventive role in dementia. However, more epidemiological studies are needed to clarify the association between BCG use and decreased dementia risk in the normal population. In addition, further bench studies are needed to better understand the mechanisms linking BCG and incident dementia.

## Data availability statement

The original contributions presented in the study are included in the article/Supplementary material, further inquiries can be directed to the corresponding authors.

## Author contributions

M-SW and Y-AZ designed the study and drafted the initial manuscript. CH and Y-LC performed the screening and collected data. JW and C-PG supervised data collection. All authors approved the final version of the report.
