# Supplementary material for: The role of Bacillus Calmette-Guérin administration on the risk of dementia in bladder cancer patients: a systematic review and meta-analysis
Source: Front Aging Neurosci. 2023 Aug 24;15:1243588. doi: 10.3389/fnagi.2023.1243588 (PMC10484104; doi:10.3389/fnagi.2023.1243588)
Supplement: Supplementary file 2 [file Data_Sheet_2.doc]

**1. Electronic search strategy**

All search run: May 20^th^, 2023

**2. Databases (searching syntax; records, n)**

**2.1 PubMed (n=726)**

(BCG OR bacillus OR "calmette guerin" OR "mycobacterium bovis" OR ((mycobacteri* OR tuberculosis) AND (vaccin* OR vacin* OR vaksin*))) AND ((cogniti* AND (impair* OR declin* OR deficit* OR disturb* OR disorders)) OR dementia* OR alzheimer* OR "alzheimer* disease" OR "AD")

**2.2 Embase (n=1284)**

(BCG OR bacillus OR 'calmette guerin' OR 'mycobacterium bovis' OR ((mycobacteri* OR tuberculosis) AND (vaccin* OR vacin* OR vaksin*))) AND ((cogniti* AND (impair* OR declin* OR deficit* OR disturb* OR disorders)) OR dementia* OR alzheimer* OR 'alzheimer* disease' OR 'AD')

**2.3 Scopus (n=1105)**

(BCG OR bacillus OR "calmette guerin" OR "mycobacterium bovis" OR ((mycobacteri* OR tuberculosis) AND (vaccin* OR vacin* OR vaksin*))) AND ((cogniti* AND (impair* OR declin* OR deficit* OR disturb* OR disorders)) OR dementia* OR alzheimer* OR "alzheimer* disease" OR" AD")

**2.4 Web of Science (n=882)**

(BCG OR bacillus OR "calmette guerin" OR "mycobacterium bovis" OR ((mycobacteri* OR tuberculosis) AND (vaccin* OR vacin* OR vaksin*))) AND ((cogniti* AND (impair* OR declin* OR deficit* OR disturb* OR disorders)) OR dementia* OR alzheimer* OR "alzheimer* disease" OR (AD)*)

**2.5 CINAHL (n=34)**

(BCG OR bacillus OR "calmette guerin" OR "mycobacterium bovis" OR ((mycobacteri* OR tuberculosis) AND (vaccin* OR vacin* OR vaksin*))) AND ((cogniti* AND (impair* OR declin* OR deficit* OR disturb* OR disorders)) OR dementia* OR alzheimer* OR "alzheimer* disease" OR AD)

**2.6 Cochrane Library (n=12)**

(BCG OR bacillus OR "calmette guerin" OR "mycobacterium bovis" OR ((mycobacteri* OR tuberculosis) AND (vaccin* OR vacin* OR vaksin*))) AND ((cogniti* AND (impair* OR declin* OR deficit* OR disturb* OR disorders)) OR dementia* OR alzheimer* OR (alzheimer* NEXT "disease"))

Supplementary instruction: The search term "AD" generated too many no related literature, so deleted.
